# Supplementary material for: Human Epidemiology and RespOnse to SARS-CoV-2 (HEROS): Objectives, Design and Enrollment Results of a 12-City Remote Observational Surveillance Study of Households with Children using Direct-to-Participant Methods
Source: medRxiv. 2022 Jul 10:2022.07.09.22277457. Preprint. [Version 1] doi: 10.1101/2022.07.09.22277457 (PMC9298141; doi:10.1101/2022.07.09.22277457)
Supplement: Supplement 1 [file media-1.pdf]

Supplementary Materials

Human Epidemiology and RespOnse to SARS-CoV-2 (HEROS): Objectives, Design and Enrollment Results of a 12-City Remote Observational Surveillance Study of Households with Children using Direct-to-Participant Methods

Contents

Supplemental methods……………………………………………………………………2

Supplemental tables……………………………………………………………………4

Supplemental figures……………………………………………………………………9

Participant surveys link…………………………………………………………………13

Supplemental methods

Study objectives

The primary objective for HEROS was to determine the incidence of SARS-CoV-2 infection via detection of viral RNA in nasal secretions. Secondary objectives included: (1) to determine SARS-CoV-2 antibody development in children and their household members, (2) to compare SARS-CoV-2 infection status and virus-specific antibody development in children with asthma and other atopic conditions (e.g. eczema) versus children without asthma or other atopic conditions, (3) to determine the mucosal immune response to SARS-CoV-2 infection through gene expression profiling and to examine whether it is influenced by the presence of asthma and other atopic conditions, (4) to determine whether topical, systemic, or inhaled steroid use, as directed by the participants' health care provider, modifies the risk of SARS-CoV-2, the severity of infection, or the expression of the SARS-CoV-2 receptor, (5) to determine whether baseline demographic and environmental factors modify the risk of SARS-CoV-2 infection or the severity of infection, and (6) to assess whether a history of respiratory syncytial virus infection or bronchiolitis during infancy modifies severity of SARS-CoV-2 infection.

Participants and enrollment

An eligible household had to include a 21-years-old and younger individual who was or had been a participant in a NIH-funded asthma and other allergic diseases study (principal participant) and who was expected to remain in the United States with a caregiver for the duration of the study. In a few rare instances, individuals that had been participants in a NIH-funded allergic disease cohort and had aged into adulthood was enrolled in HEROS as a caregiver and their children were enrolled as the principal participant. Only individuals who had previously agreed to be contacted for other research were approached.

Biospecimen collection and tracking

All materials needed to collect and return a nasal swab, blood or stool collection for a household were provided in an individual kit boxes labeled on the outside by collection type. These boxes were customized depending on whether there were 2, 3 or 4 household members enrolled in the study. The initial collection box included a welcome letter for participants, instructions for sample collection, a color-coding chart for sample labeling and explanation of the kit contents. Supplies for the baseline nasal swab, blood and stool collections for the household, as well as nasal swab collection boxes for the next 3 collection timepoints were included. Also, a "Sick Kit" that included additional supplies for nasal and stool collections in the event a person in the family were to meet the criteria for an illness event requiring additional sampling. After getting some feedback from participants, the lead site study coordinator created an Unboxing Video where she showed the contents of each box and explained how to use them. A QR code linking to this video was included on the welcome letter and in the data entry system.

Three separate shipments were sent to households containing the nasal swab boxes as well as supplies for blood collection with the Tasso device and stool collection. A color-coding system was utilized to assign a specific color to each study participant in the household and color charts were provided to the households to assist with sample tracking. Each tube was labeled with a barcode and the color was typed on the label. Participants were instructed to take a picture of their samples with barcodes facing up. The barcodes were entered by the participant and the image of the samples were uploaded to the REDCap database. The images were used during the database clean up to reconcile barcode numbers entered by participants and samples that arrived at the biorepository. As the Tasso blood collection kits were validated in their shipping boxes, they were unable to be labeled directly on the tube by the supply

vendor. The vendor provided a label in a zipped bag taped to the Tasso box. Participants packaged their samples in shipping materials provided and were instructed to mail the same or the next day via USPS.

Participants were instructed to swab the anterior nares for participants 10 years and younger and the interior turbinate for participants 11 years and older. If a study-specified symptom threshold was met (Supplemental Figure 1), an illness event was triggered for the household and additional illness-associated samples from all enrolled household participants were collected. At the time an illness event was triggered, an additional Tasso blood collection device would be shipped separately. If a household member not enrolled in HEROS developed symptoms consistent with COVID-19, the affected household member was provided the opportunity to review the consent-like study information sheet and enroll in the study to provide demographic, medical history, current medication, and the illness-associated samples.

Data collection instruments

The source instrumentation and corresponding items for HEROS baseline questionnaire and surveys were selected based on their relevance to the HEROS objectives and overall performance and/or extent of use when previously fielded. Minor modifications were made to items to ensure consistency in language or relevance to the study objectives. In addition, the baseline questionnaire and surveys contain some supplementary items that were developed by the study team based on review of COVID-19 relevant literature and consultation with study collaborators. The enrollment survey collected information on basic demographics and household features, and the enrollment and biweekly surveys included assessment of factors associated with exposure risks and risk-taking behaviors. A list of exposures was asked at the household level at enrollment and biweekly. The enrollment health assessment included: health history (allergies, allergies to food, hay fever/allergic rhinitis, asthma, autoimmune conditions, cancer, COPD, congestive heart failure, coronary artery disease, cystic fibrosis, emphysema, eczema (atopic dermatitis), heart attack, high cholesterol, hypertension, influenza, peripheral vascular disease, pneumonia, sleep apnea, type 1 or type 2 diabetes), current pregnancy, weight, height, quantified alcohol use, quantified smoking, detailed medication use, and home environment (number of household members, number of bedrooms, type of dwelling, pets). These included: Travel outside of the city or town that the family lives in, in-person work, in-person school, daycare, grocery store, in-person healthcare appointment, going out to eat, spending time with friends or family that do not live in the household, gatherings such as church or concerts, bringing home take-out food.

For those households that indicated a preference for communication via text or email, a link to the electronic surveys was sent to CG1 every week. A reminder was sent 24 and 48 hours later if the surveys were not completed. CG1 had 6 days to complete a survey before it was closed.

Supplemental Table 1. Asthma and allergic disease focused cohorts included in HEROS, research cores, and associated funding

Cohort name	Area/region	Type of cohort or study site	Grant numbers
CAS	Southeastern MI, Midwestern US	Population-based cohort	AI024156<br>AI051598<br>UG3OD023282
CCAAPS	Southwestern OH, Midwestern US	High risk cohort	3U19AI070235-14S1
CEGIR – Cincinnati	Southwestern OH, Midwestern US	Disease specific cohort - Eosinophilic gastrointestinal disorder	3U54AI117804-06S1<br>3U54AI117804-07S1
CEGIR – Denver	Central CO, Rocky Mountains US	Disease Specific cohort - Eosinophilic gastrointestinal disorder	3U54AI117804-06S1<br>3U54AI117804-07S1
CHIME	Northeastern MA, Northeastern US	Convenience cohort	R01AI127507
COAST	Southwestern WI, Midwestern US	High risk cohort	U19 AI104317<br>PO1HL70381
EASY	Northeastern MA, Northeastern US	Population-based and high risk cohort	U01 AI 110397<br>R01 HL 137192<br>K24 AI 106822<br>U10 HL109172
FORWARD	Northeastern IL, Midwestern US	Disease specific cohort – Food allergy	3R01AI130348-04S1
GCPCR	Southwestern OH, Midwestern US	Convenience cohort	3U19AI070235-14S1
ICAC – Boston	Northeastern MA, Northeastern US	Convenience cohort; Disease specific cohort – Asthma; High-risk birth cohort – Children known to be at risk for developing asthma or allergic disease	1UL1TR001430<br>5UM1AI114271
ICAC – Cincinnati	Southwestern OH, Midwestern US	Convenience cohort; Disease specific cohort – Asthma	3UM1AI114271-06S1<br>3UM1AI114271-07S1
ICAC – Dallas	Central TX, Southwestern US	Convenience cohort; Disease specific cohort – Asthma	5UM1AI114271
ICAC – Denver	Central CO, Rocky Mountains US	Convenience cohort; Disease specific cohort – Asthma	5UM1AI114271
ICAC – Detroit	Southeastern MI, Midwestern US	Convenience cohort; Disease specific cohort – Asthma	UM1AI114271
ICAC – New York	Metro NY, Northeastern US	Convenience cohort; Disease specific cohort –	3UM1AI114271-06S1<br>5UM1AI114271

		Asthma; High-risk birth cohort – Children known to be at risk for developing asthma or allergic disease	
ICAC – St. Louis	Eastern MO, Midwestern US	Disease specific cohort – Asthma; High-risk birth cohort – Children known to be at risk for developing asthma or allergic disease	3UM1AI14271-07S1<br>UM1 AI11427
ICAC – Washington D.C.	Southwestern US	Convenience cohort; Disease specific cohort – Asthma	5UM1AI114271
INSPIRE	Middle TN, Southeastern US	Population-based birth cohort	U19 AI 095227-S2<br>U19 AI 095227- S1
iTRACC	Northeastern IL, Midwestern US	Disease specific cohort – Asthma	3R01AI130348-04S1
MAAP	Southeastern MI, Midwestern US	Population-based birth cohort	3PO1AI089473-07S1<br>AI089473<br>UG3OD023282
MPAACH	Southwestern OH, Midwestern US	Disease specific cohort – Atopic dermatitis	3U19AI070235-14S1
National Jewish Health	Central CO, Rocky Mountains US	N/A	NIH 3UM1AI151958-01S1<br>NIH 3UM1AI151958-02S1
Rho	Eastern NC, Southeastern US	N/A	1UM2AI117870
SARP	Northeastern MA, Northeastern US	Disease specific cohort – Asthma	U01 AI 110397<br>R01 HL 137192<br>K24 AI 106822<br>U10 HL109172
SICAS	Northeastern MA, Northeastern US	Disease specific cohort – Asthma	U01 AI 110397<br>R01 HL 137192<br>K24 AI 106822<br>U10 HL109172
Vanderbilt Coordinating Center	Middle TN, Southeastern US	Data coordinating center	U19 AI 095227- S1
WHEALS	Southeastern MI, Midwestern US	Population-based birth cohort	AI050681<br>AI089473<br>UG3OD023282
WIND	Northeastern MA, Northeastern US	Disease specific cohort - severe (hospitalized) bronchiolitis during infancy	R01AI127507
WISC	North Central WI, Midwestern US	Children enrolled from farm families and non-farm families	U19 AI104317<br>UH3 OD023282

Supplemental Table 2. Self-reported household clinical and social characteristics at enrollment

	Completed, N = 3,081[1]	Withdrawn, N = 2,517[1]	Overall, N = 5,598[1]	p-value[2]
**Doctor Indicated COVID**	37 (1.2%)	37 (1.5%)	74 (1.3%)	0.4
**Allergies (Hay Fever, Allergic Rhinitis)**	1,383 (45%)	1,123 (45%)	2,506 (45%)	0.8
**Allergies to Food**	489 (16%)	395 (16%)	884 (16%)	0.9
**Asthma**	830 (27%)	843 (33%)	1,673 (30%)	<0.001
**Eczema**	555 (18%)	440 (17%)	995 (18%)	0.6
**No Reported Allergic Disease**	1,248 (41%)	965 (38%)	2,213 (40%)	0.10
**Alcohol Consumption Frequency**				<0.001
Four or more times a week	138 (9.3%)	82 (7.0%)	220 (8.3%)	
Monthly or less	433 (29%)	406 (35%)	839 (32%)	
Never	318 (21%)	311 (27%)	629 (24%)	
Prefer not to answer	20 (1.3%)	26 (2.2%)	46 (1.7%)	
Two to four times a month	296 (20%)	226 (19%)	522 (20%)	
Two to three times a week	283 (19%)	120 (10%)	403 (15%)	
N Missing	1,593	1,346	2,939	
**Smoking Cigarette/Cigar Frequency**				<0.001
Daily	67 (3.8%)	145 (10%)	212 (6.7%)	
Less than Daily	55 (3.1%)	61 (4.4%)	116 (3.7%)	
Not at all	1,627 (93%)	1,175 (84%)	2,802 (89%)	
Prefer not to answer	8 (0.5%)	13 (0.9%)	21 (0.7%)	
N Missing	1,324	1,123	2,447	
**Electronic Nicotine Product Frequency**				0.058
Daily	17 (1.0%)	21 (1.5%)	38 (1.2%)	
Less than Daily	15 (0.9%)	25 (1.8%)	40 (1.3%)	
Not at all	1,717 (98%)	1,340 (96%)	3,057 (97%)	
Prefer not to answer	8 (0.5%)	6 (0.4%)	14 (0.4%)	
N Missing	1,324	1,125	2,449	

[1]n (%)

[2]Pearson's Chi-squared test

Supplemental Table 3. Biospecimen collection adherence by caregiver demographics

	% Blood Samples mean (sd)	% Nasal Samples median (q1, q3)
**Sex**		
Female	54% (41%)	57% (10%, 86%)
Intersex	100% (NA)	64% (64%, 64%)
Male	66% (40%)	79% (43%, 93%)
Prefer Not to Answer	100% (NA)	64% (64%, 64%)
Unknown	0% (NA)	0% (0%, 0%)
**Race**		
American Indian or Alaska Native	49% (44%)	43% (0%, 75%)
Asian	70% (35%)	82% (57%, 93%)
Black or African American	37% (40%)	20% (0%, 64%)
More than One Race	48% (44%)	43% (10%, 79%)
Native Hawaiian or Other Pacific Islander	50% (71%)	55% (32%, 78%)
Prefer Not to Answer	51% (42%)	48% (0%, 80%)
Unknown	0% (NA)	0% (0%, 0%)
White or Caucasian	65% (38%)	76% (30%, 93%)
**Ethnicity**		
Hispanic	54% (42%)	50% (10%, 79%)
Non-Hispanic	55% (41%)	62% (10%, 86%)
Prefer Not to Answer	41% (41%)	15% (0%, 80%)
Unknown	0% (NA)	0% (0%, 0%)
**Smoking History**		
Daily	33% (37%)	10% (0%, 64%)
Less than Daily	43% (44%)	40% (0%, 79%)
Not at all	57% (41%)	64% (10%, 86%)
Prefer not to answer	44% (40%)	29% (0%, 52%)

Supplemental Table 4. Adverse events	
	N = 5,598[1]
**Toxicity Grade**	
No AE Reported	5,579 (99.66%)
Grade 1 (Mild)	15 (0.27%)
Grade 2 (Moderate)	1 (0.02%)
Grade 3 (Severe and undesirable)	3 (0.05%)
**Relation to Blood Collection**	
Not Related	1 (5.26%)
Possibly Related	5 (26.32%)
Definitely Related	13 (68.42%)
**Relation to Nasal Swab Collection**	
Not Related	17 (89.47%)
Possibly Related	1 (5.26%)
Definitely Related	1 (5.26%)
**Concussion**	1 (0.02%)
**Contusion**	1 (0.02%)
**Dermatitis contact**	1 (0.02%)
**Dizziness**	2 (0.04%)
**Epistaxis**	2 (0.04%)
**Facial bones fracture**	1 (0.02%)
**Syncope**	6 (0.11%)
**Vascular catheter specimen collection**	1 (0.02%)
**Vessel puncture site bruise**	3 (0.05%)
**Vessel puncture site hemorrhage**	1 (0.02%)
**Vessel puncture site hypoaesthesia**	1 (0.02%)
**Vessel puncture site pain**	2 (0.04%)
**Vessel puncture site rash**	1 (0.02%)
**Vessel puncture site swelling**	2 (0.04%)

[1]n (%)

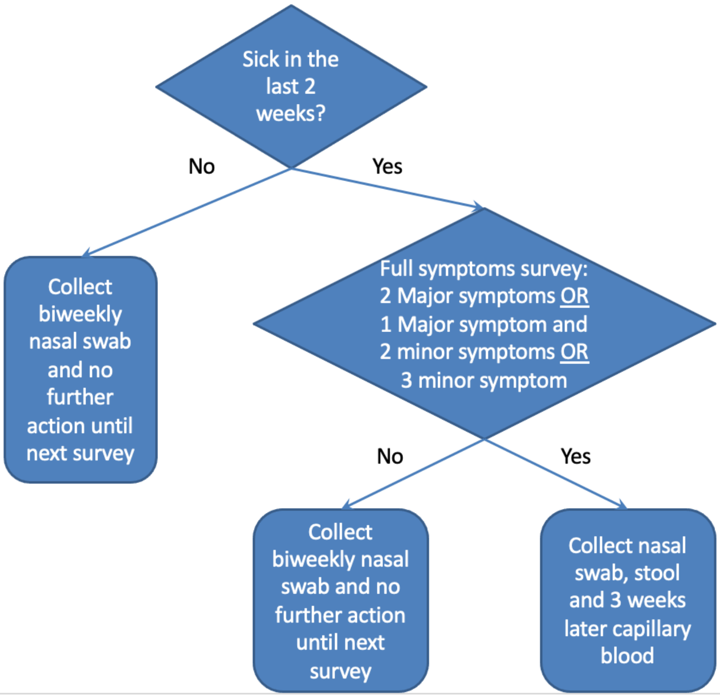

Algorithm: (Were you sick within the last 2 weeks? = YES) AND (2 Major symptoms OR 1 Major symptom and 2 minor symptoms OR 3 minor symptoms)

MAJOR SYMPTOMS
Did you have a fever or felt feverish (chills, sweating) in the last 2 weeks? =YES
Did you have a cough in the last 2 weeks? = YES
Did you have any shortness of breath at any time during the last 2 weeks= YES
Did you have any pain or discomfort in your chest within the last 2 weeks? = YES
Did you feel tired or fatigued during the last 2 weeks? = YES
Did you experience any diarrhea within the last 2 weeks? = YES

MINOR SYMPTOMS
Did you have any body aches during the last 2 weeks? = YES
Did you have any cold or flu-like symptoms (such as a sore throat, runny nose, or congestion)=YES
Did you have any headaches at any time during the last 2 weeks=YES
Did you have a problem with your ability to smell, such as not being able to smell things or things not smelling the way they are supposed to within the last 2 weeks? = YES
Did you have a problem with your ability to taste sweet, sour, salty, or bitter foods and drinks within the last 2 weeks? = YES
Did you experience any nausea (feeling you might vomit) within the last 2 weeks? = YES
Did you experience any belly pain at any time within the last 2 weeks? = YES
Did you have red or pink eyes at any time during the last 2 weeks = YES
For age < 2 years, irritability
For age < 2 years, difficulty feeding
For age < 2 years, altered sleep (more or less)
For age < 2 years, pulling at ears
For age < 6 years, Did you have any ear pain at any time during the last 2 weeks

Supplemental Figure 1. Symptom algorithm to trigger an illness event and additional household biospecimen collection based on survey question responses.

Supplemental Figure 2. Flow diagram of remote data and biospecimen management.

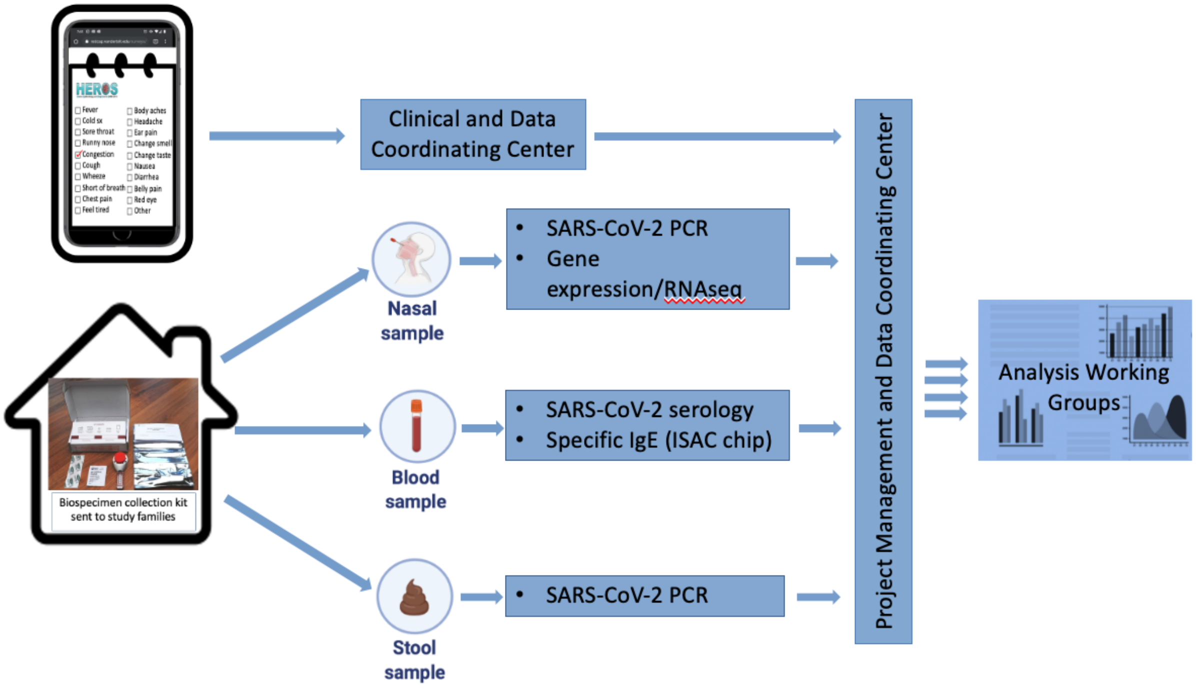

Supplemental Figure 3. Household collection of biweekly nasal samples over the study period.

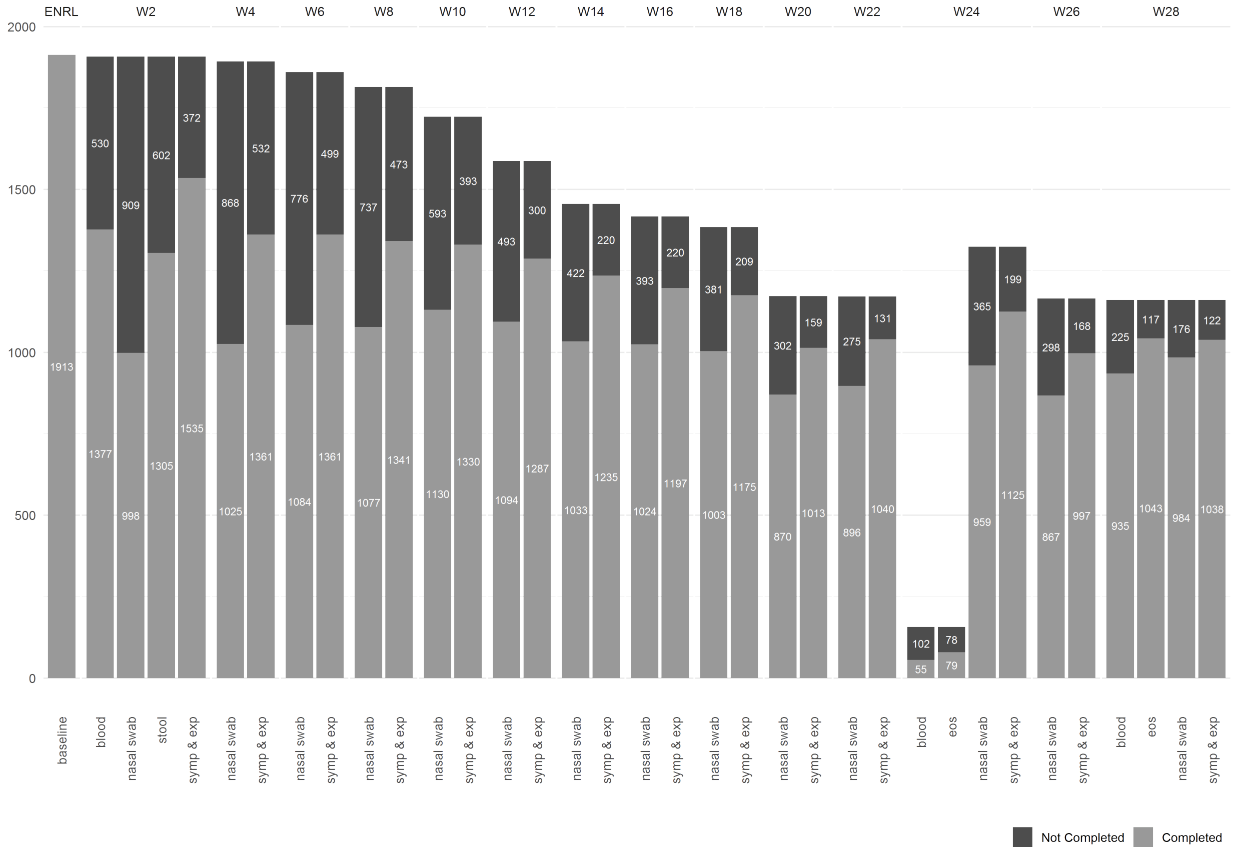

Baseline – enrollment survey
Blood – Capillary blood collection
Nasal swab – biweekly nasal collection
EOS – end of study survey
Symp & exp – biweekly survey of symptoms, behaviors and exposures

Supplemental Figure 4. Cumulative household withdrawal over study period relative to enrollment.

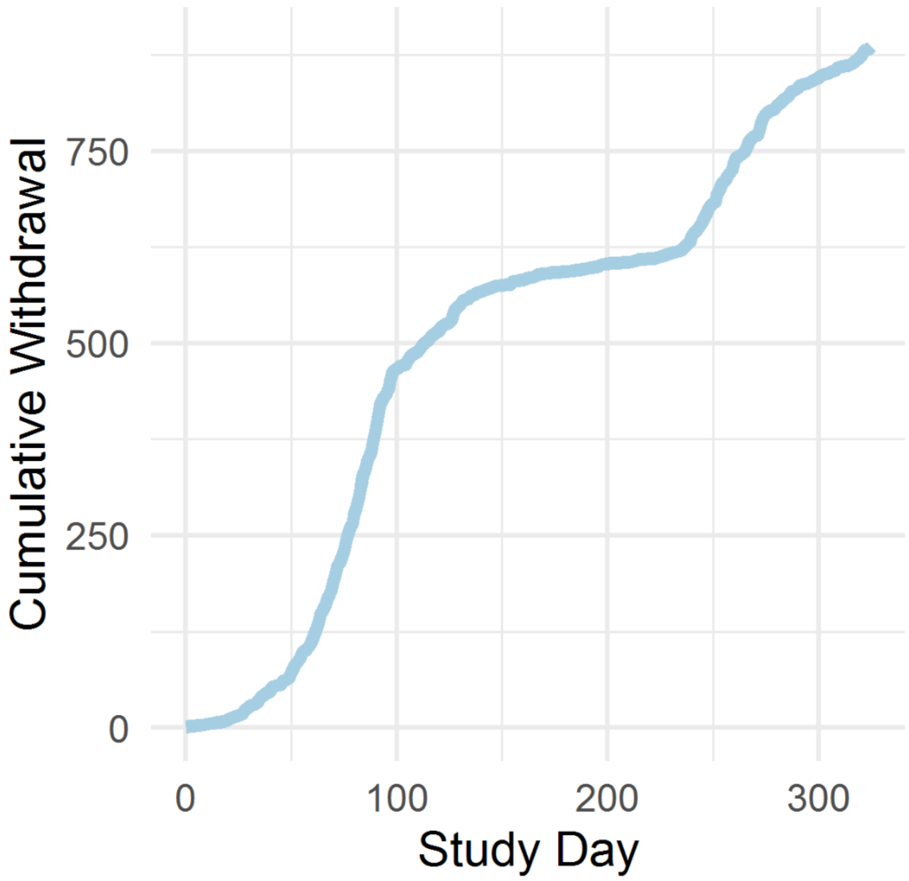

Participant Surveys Link: https://www.vumc.org/heros/survey-instruments

Downloads:

HEROS Registration Questionnaire (English)
HEROS Baseline Enrollment Questionnaire (English)
HEROS Weekly Health Check (English)
HEROS Every-Other-Week Questionnaire (English)
HEROS Child Specific Bi-Weekly Questions (English)
HEROS Sample Collection (English)
End of study survey (English)

HEROS Registration Questionnaire (Spanish)
HEROS Baseline Enrollment Questionnaire (Spanish)
HEROS Weekly Health Check Questionnaire (Spanish)
HEROS Every-Other-Week Questionnaire (Spanish)
HEROS Child Specific Bi-Weekly Questionnaire (Spanish)
HEROS Sample Collection (Spanish)
End of study survey (Spanish)
